# Asian Race and Primary Open-Angle Glaucoma: Where Do We Stand?

**DOI:** 10.3390/jcm11092486

**Published:** 2022-04-28

**Authors:** Aditya Belamkar, Alon Harris, Francesco Oddone, Alice Verticchio Vercellin, Anna Fabczak-Kubicka, Brent Siesky

**Affiliations:** 1 Department of Ophthalmology, Indiana University School of Medicine, Indianapolis, IN 46202, USA; abelamka@iu.edu; 2 Department of Ophthalmology, Icahn School of Medicine at Mount Sinai, New York, NY 10029, USA; alon.harris@mssm.edu (A.H.); alice.verticchio@mssm.edu (A.V.V.); anna.fabczak-kubicka@mssm.edu (A.F.-K.); 3 IRCCS-Fondazione Bietti, 00198 Rome, Italy; oddonef@gmail.com; 4 New York Eye & Ear Infirmary of Mount Sinai, New York, NY 10003, USA

**Keywords:** glaucoma, primary open angle glaucoma, normal tension glaucoma, Asian population, race, intraocular pressure, optic nerve head, cornea, retina, genetics

## Abstract

Primary open-angle glaucoma (POAG) is an optic neuropathy characterized by irreversible retinal ganglion cell damage and visual field loss. The global POAG prevalence is estimated to be 3.05%, and near term is expected to significantly rise, especially within aging Asian populations. Primary angle-closure glaucoma disproportionately affects Asians, with up to four times greater prevalence of normal-tension glaucoma reported compared with high-tension glaucoma. Estimates for overall POAG prevalence in Asian populations vary, with Chinese and Indian populations representing the majority of future cases. Structural characteristics associated with glaucoma progression including the optic nerve head, retina, and cornea are distinct in Asians, serving as intermediates between African and European descent populations. Patterns in IOP suggest some similarities between races, with a significant inverse relationship between age and IOP only in Asian populations. Genetic differences have been suggested to play a role in these differences, however, a clear genetic pattern is yet to be established. POAG pathogenesis differs between Asians and other ethnicities, and it may differ within the broad classification of the Asian race. Greater awareness and further research are needed to improve treatment plans and outcomes for the increasingly high prevalence of normal tension glaucoma within aging Asian populations.

## 1. Introduction

Glaucoma describes a family of optic neuropathies characterized by retinal ganglion cell loss, optic nerve head alterations, and the retinal nerve fiber layer (RNFL) thinning with associated progressive visual field loss, beginning in the peripheries. The global prevalence of the disease has been estimated to be 3.54% in populations aged 40 to 80 and 3.05% for primary open-angle glaucoma specifically (POAG) [1]. The disease is expected to afflict over 100 million individuals by 2040 [1]. Currently, intraocular pressure (IOP) is the only modifiable risk factor, with strong evidence establishing the reduction of IOP, even for those with clinically normal pressures, to significantly decrease the risk of progression [2,3]. Several risk factors for glaucoma have been identified including elevated IOP, family history, older age, and, of particular importance, race [1,3]. In fact, it has been estimated that persons of African descent may have a 2.8 times higher prevalence of POAG than those of European descent [1]. Additionally, normal-tension glaucoma (NTG), a particular type of POAG characterized by IOP within normal limits, and primary angle-closure closure glaucoma (PACG), have been established to be more prevalent in Asian populations [1]. More specifically, China and India have been predicted to have the most and second most total glaucoma cases, second and third most POAG cases, and most and second most PACG cases in the world, respectively [4]. These racial differences in risk and potential mechanistic pathways necessitate an improved understanding of glaucoma pathology to more effectively treat individuals of Asian descent. To date, the most important data available on the epidemiology of primary forms of glaucoma (including POAG with high IOP, NTG, and PACG) in Asians are from a diverse set of studies [5,6,7,8,9,10,11,12,13,14,15,16,17,18,19,20,21,22,23]; however, findings have not been well synthesized to properly evaluate the specifics of the disease within the Asian population as well as its ethnic subgroups; therefore, in this review, we will summarize the current literature on primary forms of glaucoma with an emphasis on understanding NTG and its impact in aging Asian populations.

## 2. Materials and Methods

PubMed and Embase searches were conducted for all pertinent articles and abstracts published between 1 January 2000 and 31 December 2021. Key words utilized in varying combinations include glaucoma, primary open-angle glaucoma, open-angle glaucoma, Asian, incidence, prevalence, intraocular pressure, ocular blood flow, ocular vasculature, optic nerve head, retina, retinal nerve fiber layer, cornea, central corneal thickness, and genetics. Only articles written in English were considered. Articles were screened for relevance and analyzed for inclusion in the paper by the authors. The authors first sorted through abstracts for relevance to the below outline. Abstracts that mentioned or described these topics were included for further full article review. Additionally, citations of screened articles were also reviewed and considered for inclusion based on relevance.

## 3. Results

### 3.1. Incidence and Prevalence

Asian populations, specifically of East Asian ethnic origin, have been commonly found to have the highest prevalence of and be a major risk factor for PACG [5,6,7,8]. Additionally, the pattern of disease has been described differently in Asian populations as compared to European populations, potentially due to anterior chamber and angle anatomy [8]. In comparison, studies done on POAG have been conducted in a wide variety of Asian populations, finding prevalence rates ranging from as low as 1.0% to as high as 6.5% (Table 1) [9,10,11,12,13,14,15,16,17,18,19,20,21,22,23].

Additionally, studies have estimated that approximately 70% of all POAG cases in Asians were of the normal-tension variety [13,24]. In comparison to other races, Asian populations have been found to have a relatively lower risk of POAG per decade in age, along with subtle regional differences in risk [22,25]. This risk parameter was found to be the highest in Hispanics followed by white populations; black populations had a similar risk score as Asian populations, but also had the highest prevalence at any age group [22,25]. Additionally, within Asian populations, POAG prevalence has been estimated to increase significantly in South and Central Asia, increasing from 17.06 million in 2016 to 32.09 million individuals afflicted by 2040 [11]. This increase may potentially be attributed to an aging population base or an increased access to primary care and glaucoma screening; however, further study is necessary to develop an appropriate conclusion regarding these epidemiological changes and the factors influencing them [26]. Asian populations may not have the highest prevalence percentage or increase in prevalence as other racial demographics, but their rapidly increasing population positions them to be a heavily affected group in the future. In addition to primary forms of glaucoma, secondary forms of the disease, including exfoliative and pigmentary glaucoma, exist and need further investigation in the Asian populations. Moreover, another disease highly prevalent among Asians is myopic maculopathy, a leading cause of irreversible blindness that shares a common risk factor, represented by axial elongation, with glaucoma [27]. Myopic optic neuropathy is expected to increase in the future and mostly in Asian populations, therefore, investigating the natural course of the glaucomatous disease in high myopic eyes, specifically in the Asian race, is suggested.

### 3.2. Risk Factors

Risk factors for glaucoma in Asian populations vary widely within the literature, but similarly to other racial demographics, they include: older age, family history of glaucoma, elevated IOP, thinner central corneal thickness (CCT), and myopia [28,29,30,31,32,33]. The Beijing Eye Study found glaucoma progression to be associated with smaller optic disc rim area in multiple regression analysis, but not with optic disc size, mean blood pressure, ocular perfusion pressure, retinal vessel diameter, retinal microvascular abnormalities, refractive error, and prevalence of dyslipidemia [33]. Additionally, systemic diabetes and hypertension have been implicated to increase the risk for glaucoma in Asians, which is similar to Western populations, but the role of these conditions in glaucomatous pathology is still undefined [33,34,35]. Obstructive sleep apnea and hypopnea syndrome have also been associated with an increased risk of glaucoma in Asian and Caucasian populations [36]. In addition to systemic conditions, serum levels of vitamin C and D, uric acid, and ferritin have been associated with glaucoma [37,38,39]. More specifically, lower serums levels of vitamin C and increased levels of uric acid have been associated with NTG [37]. Similarly, reduced vitamin D levels were associated, in a reverse J-shaped manner, with a significantly elevated risk of POAG [38]. Additionally, IOP and vertical and horizontal cup-to-disc ratios were significantly related to vitamin D level, potentially providing an explanation for this association [38]. In terms of social risk factors, education level has not been demonstrated to be associated with IOP or POAG in an Asian population [40]. Overall, risk factors for glaucoma may not vary between racial demographics, but further research is necessary to better understand their role in glaucoma pathology.

### 3.3. Association of Ocular Structures and Glaucoma

#### 3.3.1. Optic Nerve Head

Racial variations in the ONH and their association with glaucoma have been proposed, however, a consensus is yet to be established [41,42,43,44]. The Tanjong Pagar study considered morphometric characteristics of the ONH in a Singaporean Chinese population, and found that these parameters were incredibly similar to those described previously in other populations [41]; however, another study looking at white, Asian, African, Hispanic, and Filipino-Americans found mean optic disc sizes of 2.15 mm, 2.38 mm, 2.55 mm, 2.57 mm, and 2.48 mm, respectively, with white-Americans having significantly smaller optic discs than the other groups [42]. Mean data also suggested that Asian populations may present as a distinct intermediate between white and black patients, but this varies within subgroups [42]. Additionally, a study conducted in European Caucasian and East Asian children found that East Asian children had significantly larger mean cup-to-disc ratios [43]. Finally, significantly greater disc area, cup-to-disc area, vertical cup-to-disc area, and cup volume have been observed in Chinese populations compared with Caucasian populations [44]. Additionally, Malay patients with NTG have been found to have larger optic disc and cup areas than patients with POAG, which may further complicate its clinical utilization [45].

#### 3.3.2. Retina

Differences in retinal measurements have also been noted between Asian and Caucasian populations and within Asian ethnic groups. East Asian children have been found to have a thicker RNFL compared with European Caucasian children [43]. Additionally, a study comparing Caucasian and Chinese subjects found a significantly greater thickness in all peripapillary RNFL parameters, except for the nasal quadrant, in Chinese subjects after adjusting for age, sex, axial length, IOP, disc area, and ganglion cell complex thickness [44]; however, Caucasian subjects were found to have significantly greater ganglion cell complex thicknesses [44]. These findings indicate potential racial differences in retinal parameters, but further comparative studies are needed to appropriately draw conclusions with other racial demographics. It is important to note that studies have found similar trends in other ethnic groups. In Indian eyes, a negative correlation between age and average RNFL thickness was identified without significant differences in RNFL thickness between males and females [46]. Additionally, Indian patients with POAG were found to have significantly thinner RNFL parameters than OHT patients whereas OHT patients were found to have significantly thinner RNFL parameters than normal patients [47]. These trends have generally been identified in other ethnicities, which may thus describe commonalities in glaucoma pathogenesis in spite of population-based variations. 

Looking specifically at Asian ethnic groups, the Singapore Epidemiology of Eye Diseases Study examined normal eyes of Chinese, Malay, and Indian adults. The study found the average ganglion cell-inner plexiform layer thickness to be 82.6 ± 6.1, 81.5 ± 6.8, and 78.0 ± 6.9 μm in Chinese, Malay, and Indian participants, respectively [48]. This parameter was found to be significantly thinner in Indians compared with Chinese and Malays [48]. Additionally, the study found the average RNFL thickness to be 95.7 ± 9.6, 94.9 ± 10.6, and 87.3 ± 10.6 μm in Chinese, Malay, and Indian participants, respectively [49]. This parameter was also found to be significantly thinner in Indians compared with Chinese and Malays [49]. No statistically significant difference was observed between Chinese and Malay participants [49]. This recent, large-scale study proposes anatomical differences in key glaucomatous parameters between various Asian ethnic groups. It is essential to better identify racial differences to improve upon our understanding of glaucoma pathology as well as to develop more individualized treatment options. 

#### 3.3.3. Cornea

Although corneal measurements, particularly CCT, have been studied in a range of racial demographics, conclusions regarding Asians are still conflicted. It has been established that patients of African descent have thinner corneas whereas those of European and Latin American descent have relatively thicker corneas [50,51,52,53]. In comparison, Asians are known to fall between these extremes; however, their exact position as intermediates is yet to be established, with some studies identifying Asians as more similar to their European descent counterparts and others with their African descent counterparts [50,51,52,53]. Additionally, differences in ethnic groups have been found within the broad classification of Asian race. South and Southeast Asians, Filipinos, and Pacific Islanders have been found to have 6 to 13 µm thicker corneas than Chinese, Japanese, and Koreans [54]. Studies have identified Chinese participants to have the thinnest corneas within the Asian cohort [50,55], but a definitive conclusion has yet to be established due to similar findings in Japanese participants [53]. Additionally, ethnic variations within rural Chinese populations have been identified. A large-scale study examining 6504 adults categorized as ethnic Bai, Yi, or Han over the age of 50 years showed that ethnicity contributed significantly to the presence of thinner corneas compared with other factors including age, gender, body mass index, blood pressure, and other anterior ocular structural parameters [56]. More specifically, those of Han ethnicity were found to have the thinnest corneas [56].

Although the role of CCT in glaucoma pathogenesis is not clear, the parameter has been found to explain, at least partially, the effect of older age on increased risk of glaucoma in those of African and Latin American descent, but not in Asians [54]. The Singapore Malay Eye Study found thinner CCT to be associated with a smaller rim area and greater cup-to-disc area in POAG patients but not normal subjects, which may suggest a potential relationship between CCT and glaucomatous pathogenesis [57]. Moreover, the role of CCT in NTG compared to high-pressure POAG has yet to be understood. A Korean study found CCT to be thinner in NTG patients compared with POAG patients and control subjects [58]; however, a Chinese study found no significant difference between the CCT of NTG patients and healthy age- and gender-matched subjects [59]. Although ethnic differences may exist, further study is necessary to better understand this relationship. Greater CCT has been proposed, though, it is associated with higher IOP, younger age, male sex, non-hypertensive status, and diabetes and hyperglycemia [60,61,62,63].

#### 3.3.4. Ocular Vasculature

Retinal vascular geometry has been widely studied in Asian populations. Overall, decreased retinal arteriolar and venular tortuosity or straighter retinal vessels, narrower retinal venular branching angle, narrower retinal arteriolar and venular caliber, and decreased retinal vascular fractal dimension have been associated with an increased risk for glaucoma and progressive alterations in structural parameters including increased cup-to-disc ratio, reduced RNFL and ganglion cell-inner plexiform layer thickness, and thinner neuroretinal rim [64,65,66,67,68,69,70,71]. Interestingly, the Handan Eye Study found that both POAG and PACG patients had narrower retinal arteries and veins [71]. These findings in Asian populations have been studied in Caucasian population-based studies with conflicting results. The Blue Mountain Eye Study found that POAG eyes were much more likely to have retinal arteriolar narrowing than normal eyes [72,73]; however, the Beaver Dam Eye Study found no association between retinal vascular caliber and increased prevalence of glaucoma or larger cup-to-disc ratio [72,74]. In comparison, the Montrachet study found that decreased retinal vessel calibers were associated with decreased RNFL thickness in healthy elderly eyes [75]. Importantly, the Montrachet study also established the diagnostic ability of spectral-domain optical coherence tomography in discriminating between glaucoma patients from healthy controls, a technique which needs to also be applied to Asians and other populations to establish its diagnostic role in a clinician’s arsenal [76]. Another European study found that narrowing of both arterial and venous retinal vessels was associated with POAG [77]. The large-scale European Eye Epidemiology study noted peripapillary RNFL thickness to be associated with systemic vascular and neurovascular disease [78]. These findings may describe common mechanisms of the vascular hypothesis of glaucomatous progression; however, further research in diverse populations is required to appropriately describe the pathogenesis in both of these racial groups. 

Research on retinal vascular geometry within specific Asian ethnic groups is much more limited. One study found, through multiple linear regression modeling, that healthy Indian participants had the largest arteriolar and venular calibers and Chinese participants had the smallest vessel calibers, with Malay participants falling between these groups [79]. In addition, Chinese participants were identified to have the largest arteriolar and venular tortuosity and venular fractal dimension [79]. Both of these parameters have been previously associated with a reduced risk of glaucoma or structural progression in broader Asian studies, which may be an important consideration in understanding disease pathogenesis as it differs between ethnic groups. New developments in optical coherence tomography angiography have allowed for the collection of quantitative data on optic disc and peripapillary nerve fiber layer plexus vessel density that could serve as normative clinical references, as per a recent large-scale study conducted in Chinese adults [80]. Retinal vasculature and blood pressure differences do exist between ethnic and racial demographics and may be important in understanding the pathogenesis of glaucoma in these diverse groups.

#### 3.3.5. Other Ocular Structures

Other ocular structures have been briefly studied in Asian populations. A cross-sectional and meta-analysis study of healthy and POAG Chinese subjects found that there was no significant difference in choroidal thickness between the groups after adjusting for IOP, age, and axial length, indicating the choroid may not play a significant role in glaucoma pathogenesis, at least in this population [81]. A Korean study found CCT and anterior scleral thickness to be correlated only in NTG patients and anterior scleral thickness to be thinner in NTG patients compared with POAG patients and control subjects [58]. A study comparing iris structural measurements in American Caucasians and Chinese and mainland Chinese subjects found that Chinese subjects had thicker irises and greater iris area under dark conditions that Caucasian subjects [82]. The group also found that Chinese subjects had smaller angle recess area and trabecular-iris space area than Caucasian subjects but greater dark-to-light changes in angle opening distance and trabecular-iris space area [83]. Another study found that the lamina cribrosa thickness to be reduced in the glaucomatous group compared with the normal group [84]. This finding supports the mechanistic hypothesis postulating the role of pressures in damaging posterior ocular structures and influencing visual function. Furthermore, in healthy Asian eyes, a greater lamina cribrosa depth was found to be associated with age, the female gender, Indian race, axial length, retinal nerve fiber layer thickness, choroidal thickness, vertical cup-to-disc ratio, and disc size [85]. Although these measurements may not be directly related to the pathogenesis of glaucoma, they are still important for a better understanding of the disease and racial differences in the eye as a whole.

### 3.4. Intraocular Pressure

Due to the relatively higher incidence of NTG in Asian populations, it is thought that these patients may have a lower IOP than other racial groups more commonly afflicted with high-pressure POAG. This, at least on the surface, goes against the popular mechanistic hypothesis regarding glaucomatous progression and thus must be evaluated carefully; however, a definite conclusion regarding racial variations in IOP is difficult to establish [51,52]. A study comparing aqueous humor dynamics between Chinese and Caucasian adults found that Caucasians had lower IOP, a slower aqueous flow rate, and a faster uveoscleral outflow rate [86]. Ethnic differences may also exist in IOP, with East Asians having been suggested to have the lowest IOP [55]. The Singapore Epidemiology of Eye Diseases Study found the mean IOP to be 14.3 ± 3.1, 15.3 ± 3.7, and 15.8 ± 2.9 mmHg in Chinese, Malay, and Indian participants, respectively, with multivariate regression analysis suggesting Chinese participants to have significantly lower IOP [55]. Additionally, the prevalence of study participants with elevated IOP, defined as 21 mm Hg or greater, was found to be 2.6%, 6.2%, and 4% in Chinese, Malay, and Indians [55]. It has been suggested that this variation may be highly heritable by a large-scale Korean study. Additive genetics was found to estimate 36% of the total variance in the IOP phenotype, whereas a unique environment explained the remaining 64% [87]. Additionally, a child’s risk of having high IOP was almost 10 times greater if they have parents with high IOP [87]. Another study conducted in Korean and Mongolian populations found higher heritability estimates of approximately 50% [88]. Although IOP may display significant individual variation, it is important to consider racial and ethnic variations when utilizing target IOPs as treatment goals due to potential genetic predispositions.

Similar to other racial demographics, elevated IOP is known to increase the risk of glaucomatous progression and has been associated with a number of ocular and systemic risk factors including the female sex, thicker central corneal thickness, high myopia, high body mass index, high blood pressure, diabetes, and hyperlipidemia [88,89,90,91,92,93,94,95]; however, rather surprisingly, older age has not been associated with increased IOP in Asian populations. Instead, IOP has repeatedly been found to significantly decrease with age in this racial demographic [88,89,92,96,97,98]. A positive association between age and IOP has been previously established in white and black populations by the Beaver Dam Eye Study and Barbados Eye Study [99,100]. This racial discrepancy in IOP is incredibly significant for clinicians to consider as IOP reduction is currently the only therapy for preventing or limiting disease progression. Asian glaucoma patients may therefore display artificial reductions in IOP as they age, which is problematic as epidemiologic studies have indicated an increased risk of glaucoma with age in this population, complicating disease management as current medical therapies are focused on IOP reduction [25]. New treatment modalities may thus be vital to adequately manage glaucomatous progression in Asian populations. Theories for this discrepancy have been proposed, but further research is absolutely necessary to better understand these varied patterns to effectively treat patients of different racial backgrounds and to better understand this family of pathomechanisms classified as POAG.

### 3.5. Blood and Perfusion Pressures

Although hypertension and elevated blood pressure parameters have frequently been associated with glaucoma in those of European and African descent, their role in Asian populations is not as widely established, potentially due to the relatively lower incidence of these cardiovascular conditions in these populations [101]; however, the study of these vascular factors is incredibly important as it may provide a potential explanation, through the vascular hypothesis, for the differences in glaucomatous progression noted between Asian populations and other racial groups. The Beijing Eye Study found no significant association between arterial hypertension, blood pressure parameters, ocular perfusion pressure (OPP), and POAG progression or prevalence [33,102]; however, other studies have identified an elevated prevalence of glaucoma, particularly NTG, in hypertensive patients [103,104]. In fact, a study conducted in Chinese subjects with systemic hypertension found that blood pressure was negatively correlated with a range of RNFL thickness parameters and positively correlated with mean IOP [104]. Interestingly, the Singapore Malay Eye Study found low diastolic blood pressure, mean OPP, and diastolic OPP to be independent risk factors for POAG [105]. Additionally, the Singapore Epidemiology of Eye Diseases Study found that both low and high levels of systolic OPP, but not mid-range levels, were associated with an increased risk for POAG [106]. Studies comparing the role of hypertensive status and OPP between racial demographics were not identified. A Korean study of healthy participants found little difference between pulsatile ocular blood flow between Koreans and Caucasians [107]. Studies do indicate that ocular and systemic blood pressures may be important in the pathogenesis of POAG in Asian populations as well.

### 3.6. Genetics

The heritability of glaucoma has been widely studied in Asian populations, and many genes have been implicated to play a role in its pathogenesis. Several of the structural parameters involved in glaucomatous progression have been found to be highly heritable, with one Chinese twin study identifying approximately 80% of phenotypic variations in the optic disc to be determined genetically [108]. The study found that the correlation coefficients of heritability for disc area, cup disc, and cup-to-disc ratio were 0.79, 0.83, and 0.80 in monozygotic twin pairs and 0.30, 0.37, and 0.35 in dizygotic twin pairs, respectively [108]. Additionally, the literature suggests that less than one-tenth of POAG cases in the general population may be caused by specific genetic mutations, and most are instead explained by polygenic alterations [109]. Racial differences in genetic polymorphisms have been identified, however, the significance of these in explaining population-based differences in disease presentation has yet to be fully understood [110,111]. In fact, one multi-ethnic genome-wide meta-analysis including 34,179 cases and 349,321 controls identified 127 significant POAG loci across Europeans, Africans, and Asians [110]. It also found moderately high cross-ancestry concordance of loci involved in POAG, but also many racial-specific loci [110]. Important genes noted across ethnicities include SVEP1, RERE, VCAM1, ZNF638, CLIC5, SLC2A12, YAP1, MXRA5, and SMAD6 [110]. Another study found that European, American, and South Asian populations may share similar genetic heatmap patterns for single nucleotide polymorphisms of risk alleles for POAG, whereas African, East Asian, and Korean populations each have a distinct pattern [111]. In addition, similar findings regarding the heritability and polygenic nature of PACG have been identified, however, very different gene loci have been implicated in this disease compared with POAG [112,113]. The most common POAG genes studied in Asian populations include populations include MYOC, OPTN, CYP1B1, CAV1-CAV2, TGFBR3, ATOH7, CDKN2B/CDKN2B-AS1, SIX6, MMP, LOXL-1, TP53, TNF, APOE, TLR4, NFT4, WDR36, IL-1, and VAV2-VAV3, but numerous other loci have been noted (Table 2). Overall, genetic differences between populations are expected, and they do exist, but their role in elucidating racial differences has yet to be uncovered.

## 4. Discussion

The risk of glaucoma within aging Asian populations has never been higher or in more need of targeted research. Although African and European descent populations have been compared and contrasted to some degree, Asian populations and their specific differential glaucoma risk and disease profiles are not as well understood outside of a large number of NTG patients expected within these populations. Asians are a rapidly growing population and will account for a majority of glaucoma cases in the near future. Both PACG and NTG are known to be more prevalent in this group, but it is not yet definitively known why this may be the case. Structural parameters including the optic disc, retina, and cornea have shown both racial and ethnic differences across populations. It is not yet known whether these variations may predispose populations to glaucoma or if they are secondary to other population-based variations. Regardless, these structural differences are clinically noteworthy and may impact diagnostic standards in different racial and ethnic demographics. 

Patterns in IOP and relevant parameters have also shown important differences. Aqueous humor dynamics have been indicated to differ between Asian and Caucasian populations. More significantly, in Asian populations, older age has been associated with decreased IOP, which conflicts with previous findings of a positive association in African and European descent populations. With medical therapies for glaucoma limited to IOP reduction, lowering IOP may not be as effective in aging Asian populations given their tendency to present with NTG and a decreasing IOP with age. Novel modalities of treatment may thus be necessary to effectively manage glaucoma in this population.

Systemic blood pressure and OPP have both been specifically identified to be risk factors for glaucoma in Asians, as well as other, populations. Additionally, retinal vascular geometry has been studied in detail, with narrower retinal vessel calibers, decreased tortuosity, and decreased fractal dimension having been associated with glaucoma and parameters of progression. It is still unclear whether these findings differ from other racial demographics, as ocular blood flow parameters have been studied in greater detail in white and black populations. Further studies focused on ocular blood flow in Asian populations are necessary, as well as studies on vascular geometry in other populations. Finally, distinct genetic patterns have been identified between races; however, the extent of these similarities and differences has yet to be elucidated along with associations of genetic polymorphisms and definitive pathomechanisms. Further studies are needed to specifically investigate glaucoma genes, which are commonly shared both in Asian and other races, and genes unique to Asian populations. It is important to note that other factors may interact with specific genetic patterns and influence the disease pathophysiology, specifically in Asian ethnicities, including environmental conditions and dietary habits. Asians make up a very large portion of the global population and will soon make up the majority of glaucoma-afflicted population. With a significant impact expected both within the communities and health care systems, studies are needed to investigate mechanisms driving the different types of glaucoma within the Asian population. Focus should be placed not only on POAG forms with both high and low IOP, but also within PACG and secondary forms (such as exfoliative glaucoma, pigmentary glaucoma, and myopic optic neuropathy). Looking forward, increased awareness for clinicians and patients are important to help mitigate the outsized POAG and NTG burden within aging Asian populations, and further targeted research is needed to understand specific risk factors and pathophysiologic mechanisms driving different glaucoma types (primary and secondary) in the Asian race. A greater understanding of ocular structure, including cup-to-disc biomarkers and their differences to Western populations along with a better understanding of IOP and vascular dynamics is needed, with a particular emphasis on accounting for both genetical and environmental factors.

## Figures and Tables

**Table 1:** Prevalence and Incidence of Glaucoma in Asian Populations. POAG: Primary open-angle glaucoma; NTG: normal-tension glaucoma.

Author	Population	Prevalence/Incidence of Glaucoma	Prevalence/Incidence of POAG	Prevalence/Incidence of NTG
Stein et al., 2011 [9]	Asian Americans		6.52%	0.73%
Baskaran et al., 2015 [10]	Chinese	4.0%	1.7%	
Chan et al., 2016 [11]	Asians	3.54%	2.34%	
Guo et al., 2021 [12]	Laos	1.54%	0.62%	
Zhao et al., 2018 [13]	Chinese		2.0%	1.0%
He et al., 2015 [14]	Chinese		2.85%	
Sales et al., 2014 [15]	Filipino		11.9%	6.8%
Rauf et al., 2013 [16]	Indian		1.0%	
Narayanaswamy et al., 2013 [17]	Indian	1.95%	1.25%	
Rosman et al., 2012 [18]	Singapore Malay	4.6%	3.2%	
Liang et al., 2011 [19]	Chinese		1.0%	
Shen et al., 2008 [20]	Singapore Malay	3.4%	2.5%	
He et al., 2006 [21]	Chinese		2.1%	
Rudnicka et al., 2006 [22]	Asian		1.4%	
Koh et al., 2021 [23]	Indian	1.68%	1.37%	

**Table 2:** Genetic Findings in Adult Asian Populations. NTG: normal-tension glaucoma; PACG: primary angle-closure glaucoma; PCG: primary congenital glaucoma; POAG: primary open-angle glaucoma; HTG: high-tension glaucoma.

	Race/Ethnicity	Genetic Change	Associated Type of Glaucoma
***MYOC***			
Fan et al., 2020 [114]	Chinese	c.622G > T, p.D208Y	POAG
Lei et al., 2019 [115]	Chinese	c.1309T > C, p.Y437H	POAG
Yang et al., 2015 [116]	Chinese	c.761C < G, p.P254R	POAG
Guo et al., 2015 [117]	Asian	rs12035719, rs2075648	No association with POAG
Jin et al., 2015 [118]	Han Chinese	rs183532	PACG
Cai et al., 2012 [119]	Chinese Uygur	c.1151A > G, p.D384G	POAG
Chen et al., 2011 [120]	Chinese	c.1099G > A, p.G367R	POAG
Qu et al., 2010 [121]	Chinese	c.1084G>-	POAG
Jia et al., 2009 [122]	Northern Chinese	p.Val53Ala	POAG
Xie et al., 2008 [123]	Chinese	c.38C > T, p.Pro13Leu; c.1009C > del, p.Gln337Stop	POAG
Megkegale et al., 2008 [124]	Japanese	c.297G > C, p.Gln297His; c.363G > A, p.Ala363Thr	POAG
Kumar et al., 2007 [125]	Indian	p.Gln48His	POAG
Funayama et al., 2006 [126]	Japanese	c.227G > A, p.Arg76Lys	No association with POAG
Chakrabarti et al., 2005 [127]	Indian	c.144G > T, p.Gln48His	POAG, PCG
Ishikawa et al., 2004 [128]	Japanese	c.1105T > C, p.Phe369Leu; c.1079T > A, p.Ile360Asn; c.1087G > A, p.Ala363Thr; c.1342A > C, p.Thr448Pro	POAG
Kanagavalli et al., 2003 [129]	Indian	c.1099G > A, p.Gly367Arg; c.1130C > T, p.Thr377Met	POAG
Mukhopadhyay et al., 2002 [130]	Indian	c.144G > T, p.Gln48His; c.1109C > T, p.Pro370Leu	POAG
***OPTN***			
Cheng et al., 2010 [131]	Asian	T34T	POAG
Xiao et al., 2009 [132]	Chinese	c.1274A > G, p.Lys322Glu	POAG
Kumar et al., 2007 [125]	Indian	c.915C > G, p.Thr202Arg	POAG
Ayala-Luge et al., 2007 [133]	Asian	M98K	NTG
Sripriya et al., 2006 [134]	Indian	M98K; IVS7 + 24G > A	POAG
Funayama et al., 2004 [135]	Japanese	c.412G > A, p.Thr34Thr; c.603T→A, p.Met98Lys	POAG; NTG
Leung et al., 2003 [136]	Chinese	E103D; H486R; V148V; IVS13 + 21C > G	POAG
***CYP1B1***			
Gong et al., 2015 [137]	Chinese	p.P93S; p.R259C; p.A295T; p.L475P	POAG
Dong et al., 2012 [138]	Asian	rs180040, rs1056836, rs10012, rs1056827, rs1056837, rs2567206	No association with POAG
Chen et al., 2011 [120]	Chinese	g.17120037A > G; g.17120090C > G; g.17120026T > C	POAG
Bhattacharjee et al., 2008 [139]	Indian	c.1666G, Leu432Val	POAG
Kumar et al., 2007 [125]	Indian	c.578C > T, p.Pro193Leu; c.685G > A, p.Glu229Lys; c.875T > A, p.Met292Lys; c.1103G > A, p.Arg368His	POAG
***CAV1-CAV2***			
Huang et al., 2019 [140]	Chinese	rs548030386	Intraocular pressure
Kim et al., 2015 [141]	Korean	minor allele G of rs17588172	HTG
Huang et al., 2014 [142]	Asian	rs4236601[A]	POAG
Kato et al., 2013 [143]	Japanese	minor allele G of rs1052990	NTG
***TGFBR3***			
Chai et al., 2020 [144]	Indian, Malay, Chinese	rs1192415	Optic disc parameters
Li et al., 2015 [145]	Asian	rs1192415	POAG
Khor et al., 2011 [146]	Indian, Malay	rs1192415	Optic disc parameters
***ATOH7***			
Chai et al., 2020 [144]	Indian, Malay, Chinese	rs1900004	Optic disc parameters
Mabuchi et al., 2015 [147]	Japanese	rs1900004	POAG
Chen et al., 2012 [148]	Chinese	rs61854782, rs3858145	NTG, HTG
Khor et al., 2011 [146]	Indian, Malay	rs7916697	Optic disc parameters
***CDKN2B/CDKN2B-AS1***			
Chai et al., 2020 [144]	Indian, Malay, Chinese	rs1360589	Optic disc parameters
Hu and He, 2017 [149]	Asian	rs1063192	POAG
Mabuchi et al., 2015 [147]	Japanese	rs1063192	POAG
Nakano et al., 2012 [150]	Japanese	rs523096:A; rs518394:C; rs564398:A; rs7865618:A	POAG, NTG
Osman et al., 2012 [151]	Japanese	rs1063192	POAG
Takamoto et al., 2012 [152]	Japanese	rs523096	NTG
***SIX6***			
Chai et al., 2020 [144]	Indian, Malay, Chinese	rs33912345	Optic disc parameters
Lu et al., 2019 [153]	Asian	rs10483727, rs33912345, rs12436579	POAG
Rong et al., 2019 [154]	Chinese, Japanese	rs10483727, rs33912345, rs12436579	POAG
Mabuchi et al., 2015 [147]	Japanese	rs10483727	POAG
Osman et al., 2012 [151]	Japanese	rs10483727	POAG
***MMP***			
Zhao et al., 2020 [155]	Chinese	rs2250889; rs3918242; rs17576	PACG; POAG; both
He et al., 2017 [156]	Asian	rs1799750	POAG, PACG
***LOXL-1***			
Wu et al., 2015 [157]	Asian	rs1048661, C allele of rs2165241	POAG
Sun et al., 2014 [158]	Asian	rs2165241, rs1048661, rs3825942	No association with POAG
***TP53***			
Zhang and Wang, 2019 [159]	Chinese	rs4938723, rs1042522	POAG
Gupta et al., 2018 [160]	Indian	P72R	PACG; No association with POAG
Guo et al., 2012 [161]	Asian	Arg72Pro, intron 3 16-bp insertion	POAG
Fan et al., 2010 [162]	Chinese	R72P; rs1042522	NTG
***TNF***			
Passan et al., 2019 [163]	North Indian	c.-308G > A, c.-863C > A	POAG
Wang et al., 2012 [164]	Chinese	(-863)A allele	POAG
Fan et al., 2010 [162]	Chinese	-308G > A; rs1800629	HTG
***APOE***			
Guo et al., 2015 [117]	Asian	rs405509, rs769446, rs449647	No association with POAG
Wang et al., 2014 [165]	Asian	ε4ε4 genotype	POAG
Jia et al., 2009 [122]	Northern Chinese	−491A > T, −427T > C, −219T > G, c.526C > T for ε2, c.388T > C for ε4	No association with POAG
Lam et al., 2006 [166]	Chinese	−219T > G; −427T > C	NTG and HTG; NTG
***TLR4***			
Takano et al., 2012 [167]	Japanese	rs10759930, rs1927914, rs1927911, rs12377632, rs2149356, rs7037117	POAG, NTG
Chen et al., 2012 [168]	Southern Chinese	rs7037117	POAG
Suh et al., 2011 [169]	Korean	rs10759930, rs1927914, rs1927911, rs12377632, rs2149356, rs11536889, rs7037117, rs7045953	No association with NTG
***NTF4***			
Chen et al., 2012 [170]	Chinese	c.453G > A, p.Pro151Pro; c.470G > C, p.Gly157Ala; c.545C > T, p.Ala182Val	POAG
Rao et al., 2010 [171]	Indian	c.263C > T, p.A88V; c.453G > A, p.P151P; c.790T > G, 3′UTR; c.811G > A, 3′UTR	No association with POAG, PACG
Vithana et al., 2010 [172]	Chinese	c.338T > C, p.Leu113Ser	POAG
***WDR36***			
Lee et al., 2010 [173]	Mongolian	No specific polymorphism studied	Heritability of intraocular pressure
Jia et al., 2009 [122]	Northern Chinese	IVS5 + 30C > T	No association with POAG
Fan et al., 2009 [174]	Chinese	p.I713V	HTG
Miyazawa et al., 2007 [175]	Japanese	p.S664L; p.I264V; c.1965-30A > G	HTG
***IL-1***			
How et al., 2007 [176]	Chinese	IL1α (c.-889C > T); IL1β (c.3953C < T); IL1β (c.-511C < T)	No association with POAG, PACG
Wang et al., 2007 [177]	Chinese	IL1β c.-511; c.+3953	No association with NTG
Wang et al., 2007 [178]	Chinese	IL1α c.-889C > T	No association with NTG
Wang et al., 2006 [179]	Chinese	IL1α c.-889C > T	POAG
***VAV2-VAV3***			
Shi et al., 2013 [180]	Japanese	rs2156323, rs2801219	No association with POAG, NTG
Rao et al., 2010 [171]	Indian	rs2156323, rs2801219	No association with POAG, PACG
***Chromosome 2p16.3***			
Meng et al., 2015 [181]	Chinese	rs1533428, rs12994401, rs10202118	POAG
Chen et al., 2012 [168]	Southern Chinese	rs1533428	POAG
***ABCA1***			
Huang et al., 2019 [140]	Chinese	rs2472494	Intraocular pressure
Chen et al., 2014 [168]	Southern Chinese	rs2487032	POAG
***PMM2***			
Chen et al., 2014 [182]	Southern Chinese	rs3785176	POAG
***GLIS3***			
Li et al., 2020 [183]	Han Chinese	rs736893	POAG
Huang et al., 2019 [140]	Chinese	rs7047871	Intraocular pressure
***RAMP2***			
Gong et al., 2019 [184]	Han Chinese	p.Glu39Asp; p.Glu54Lys; p.Phe103Ser; p.Asn113Lysfs*10; p.Glu143Lys; p.Ser171Arg	POAG
***ABCC5***			
Tang et al., 2017 [185]	Chinese	rs939336, rs1132776, rs983667	PACG
Nongpiur et al., 2014 [186]	Asian	rs1401999	PACG
***HTR3D***			
Tang et al., 2017 [185]	Chinese	rs12493550	PACG
***hOGG1***			
Zeng et al., 2017 [187]	Han Chinese	p.Ser326Cys	PACG
***APE1***			
Zeng et al., 2017 [187]	Han Chinese	p.Asp148Glu	PACG
***XRCC1***			
Zeng et al., 2017 [187]	Han Chinese	p.Arg399Gln	PACG
Yousaf et al., 2011 [188]	Pakistani	c.1316G > A (rs25487)	POAG
***XPD***			
Yousaf et al., 2011 [188]	Pakistani	c.2298A > C (rs13181)	POAG
***MFRP***			
Wang et al., 2018 [189]	Northern Chinese	rs2510143, rs36015759, rs3814762	No association with PACG
Shi et al., 2013 [190]	Han Chinese	rs3814762	PACG
***ZNRF3***			
Wang et al., 2018 [189]	Northern Chinese	rs7290117, rs2179129, rs4823006, rs3178915	No association with PACG
***HGF***			
Wang et al., 2018 [189]	Northern Chinese	rs5745718, rs12536657, rs12540393, rs17427817, rs3735520	No association with PACG
***CAT***			
Gong et al., 2018 [191]	Chinese	rs769217	POAG
***GJA1***			
Huang et al., 2015 [192]	Chinese	c.791_792delAA, p.K264Ifs*43	POAG
***SOD2***			
Zhou et al., 2015 [193]	Chinese	rs6917589, rs5746136	POAG
***CD2***			
Liu et al., 2014 [194]	Han Chinese	p.Gln596Trp	POAG
***GSTM1/GSTT1***			
Lu et al., 2013 [195]	East Asian	Null genotype	POAG
Huang et al., 2013 [196]	Asian	Null genotype	POAG
***HSP70***			
Shi et al., 2013 [190]	Han Chinese	rs1043618	PACG
***OPA1***			
Guo et al., 2012 [197]	Asian	rs166850, rs10451941	No association with NTG
Woo et al., 2004 [198]	Korean	IVS8 + 4C > T; c.32T > C	No association with NTG
***SLC1A3***			
Yasumura et al., 2011 [199]	Japanese	rs13173144, rs1366632, rs1428967, rs930072, rs2301066	No association with NTG
***HLA***			
Suzuki et al., 2010 [200]	Japanese	27 HLA-DRB1 alleles, 14 HLA-DQB1 alleles	No association with NTG
***GLC1F***			
Murakami et al., 2010 [201]	Japanese	163 allele of D7S1277i	NTG
***OLFM2***			
Funayama et al., 2006 [126]	Japanese	p.Arg144Gln	POAG
***EDNRA***			
Kim et al., 2006 [202]	Korean	c.*1222C > T	NTG

## Data Availability

No new data were created or analyzed in this study. Data sharing is not applicable to this article.
